# Evaluating Weight Loss Efficacy in Obesity Treatment with Allurion’s Ingestible Gastric Balloon: A Retrospective Study Utilizing the Scale App Health Tracker

**DOI:** 10.3390/clinpract14030061

**Published:** 2024-05-06

**Authors:** Danut Dejeu, Paula Dejeu, Paula Bradea, Anita Muresan, Viorel Dejeu

**Affiliations:** 1 Surgical Oncology Department, Emergency County Hospital Oradea, Strada Gheorghe Doja 65, 410169 Oradea, Romania; ddejeu@uoradea.ro (D.D.); amuresan@uoradea.ro (A.M.); 2 Bariatric Surgery Department, Medlife Humanitas Hospital, Strada Frunzisului 75, 400664 Cluj Napoca, Romania; 3 Laboratory Medicine Unit, Betania Medical Center, Menumorut 12, 410004 Oradea, Romania; 4 Gastroenterology Unit, Betania Medical Center, Menumorut 12, 410004 Oradea, Romania; paula.bradea@betania-centrulmedical.ro; 5 Bariatric Surgery Department, Life Memorial Hospital, Calea Grivitei 365, 010719 Bucuresti, Romania; office@doctordejeu.ro

**Keywords:** obesity, physical activity, weight loss, metabolic syndrome, nutrition, gastric ballon

## Abstract

Obesity represents a growing public health concern, affecting more than 15% of the global adult population and involving a multi-billion market that comprises nutritional, surgical, psychological, and multidisciplinary interventions. The objective of this retrospective study was to evaluate the short-term efficacy and body weight measurements associated with differing levels of physical activity following the use of Allurion’s ingestible gastric balloon that was designed to increase feelings of fullness and decrease food consumption, being naturally eliminated after approximately 16 weeks. This study involved 571 individuals who qualified for the intervention for being older than 20 years with a body mass index (BMI) of 27 kg/m^2^ or more. Utilizing the Scale App Health Tracker and Allurion’s smartwatch, this study was able to track vital signs and physical activity in real time. The participants had an average initial BMI of 34.1 kg/m^2^ and a median age of 41 years. Notable outcomes were observed in both study groups, “Less Active” and “More Active”, which were classified by achieving less or more than a median number of 8000 daily steps. Specifically, body fat percentage saw a reduction from 33.1 ± 9.4 to 28.3 ± 10.2 in the less active group and from 32.2 to 27.5 in the more active group, with both groups achieving statistical significance (*p* < 0.001). Additionally, there was a significant reduction in average weight, dropping from 98.2 ± 22.8 kg to 84.6 ± 19.3 kg in the less active group and from 97.7 ± 21.0 kg to 82.1 ± 22.9 kg in the more active group (both *p* < 0.001). Interestingly, those in the more active group also experienced a significant increase in lean mass compared to their less active counterparts (*p* = 0.045), although no substantial differences in weight loss, BMI reduction, and total body fat decrease were observed between the two groups. This investigation confirms the hypothesis that Allurion’s ingestible gastric balloon significantly reduces weight in the short term and enhances several physical health metrics, demonstrating effectiveness as an autonomous method for challenging weight management, regardless of the level of daily physical activity.

## 1. Introduction

The global public health landscape is increasingly marked by a rising incidence of obesity, an epidemic that poses significant challenges to health systems worldwide [1,2]. Obesity is an established precursor to myriad health complications, including but not limited to type II diabetes, hypertension, coronary artery diseases, and certain forms of malignancies [3,4,5,6]. The difficulty with weight loss lies not merely in the initial achievement of a healthy weight but extends to the long-term maintenance of this weight loss, a process entailing rigorous lifestyle alterations and continuous adherence to them [7].

Weight loss interventions can be conventionally delineated into three main categories, including lifestyle interventions, pharmacotherapy, and surgical interventions [8,9]. Lifestyle interventions, involving comprehensive modifications in dietary habits and physical activity, form the basis of any successful weight management strategy [10]. However, they may prove insufficient for certain individuals, specifically those with severe obesity. Pharmacotherapy can supplement lifestyle modifications but is often accompanied by adverse effects, and the weight loss associated with it is usually modest and temporary [11].

Bariatric surgery emerges as a more aggressive and effective treatment modality for obesity, capable of engendering substantial, sustained weight loss [12,13,14]. Nevertheless, it is not without its disadvantages. Surgical interventions carry with them an inherent risk of complications, such as infection, postoperative bleeding, and anesthetic risks, in addition to potential long-term nutritional deficiencies and metabolic complications [15,16].

Quality of life, an often-overlooked aspect of weight management, is an essential parameter in the evaluation of any weight loss strategy as much as for other medical conditions [17,18]. The objective of weight loss intervention extends beyond mere anthropometric changes, aspiring to augment overall health status, physical functioning, and psychosocial well-being. Invasive interventions, despite their efficacy, may bear negatively on quality of life, attributable to the period of convalescence, alterations in body image, and enduring dietary restrictions post-surgery [19,20,21].

In light of these considerations, non-invasive weight loss methodologies present a promising avenue for weight management [22,23]. One such emerging technology is Allurion’s ingestible gastric balloon, which facilitates weight loss by inducing early satiety [24]. This novel device is administered orally, inflated in the stomach, and naturally excreted after four months, obviating the need for surgery, endoscopy, or anesthesia [25]. It represents an innovative strategy that could effectively lower the barriers for individuals who are in need of weight management assistance but are reticent to undergo invasive procedures [26]. Complementing these non-invasive interventions are mobile health applications like the Scale App Health Tracker [27]. In an era marked by rapid digitalization, these platforms provide a user-friendly, non-invasive, and accessible means of tracking weight loss progress [28].

Despite the bariatric intervention, physical activity plays an essential role in metabolic health and weight management outcomes [29]. Engaging in regular physical activity not only enhances caloric expenditure but also modulates metabolic efficiency, impacting the body’s response to weight loss interventions [30]. Differences in physical activity levels can lead to variations in metabolic rates, body composition, and overall energy balance, which are important for assessing the effectiveness of any weight loss treatment [31]. Moreover, many patients who need and request appointments for weight loss treatment have already been involved in weight loss interventions, such as exercise, but have failed to achieve their desired weight loss or their pre-existing condition limits their exercise capacity [32,33]. As such, it is important to determine if Allurion’s ingestible gastric balloon can exhibit similar outcomes, regardless of physical activity.

This study hypothesized that Allurion’s ingestible gastric balloon will exhibit short-term efficacy in inducing clinically significant weight loss in obese and overweight patients, regardless of their engagement with physical activity, as captured through the Scale App Health Tracker. Therefore, the primary objective of this study was to assess the short-term efficacy of Allurion’s ingestible gastric balloon in affecting total body weight loss, fat loss, and visceral fat loss. The secondary objective was to determine significant changes in total body weight loss, fat loss, and visceral fat loss based on the patients’ engagement and levels of physical activity as monitored through the Scale App Health Tracker v.1.4.

## 2. Materials and Methods

### 2.1. Study Design and Ethics

This investigation was constructed as a retrospective, observational, single-center study, following a cohort of patients who used Allurion’s ingestible gastric balloon (Allurion Technologies, Inc. Natick, MA, USA). The included patients were followed for at least 16 weeks, according to the producer’s guidelines, allowing the gastric balloon‘s natural excretion after approximately 4 months. Patients were included in this study according to the Allurion guidelines that were approved in Romania. Each participant supported the procedure costs after providing voluntary, informed consent in strict accordance with the ethical guidelines outlined by the Declaration of Helsinki, ensuring complete transparency and ethical integrity throughout this study. The participants’ consent was received before the procedures, while each physician and patient had to carefully evaluate both the risks and benefits of treatment before using Allurion’s ingestible gastric balloon.

Physicians informed potential patients about the benefits and risks of gastric balloons and Allurion’s ingestible gastric balloon before treatment. They clearly communicated all contraindications, precautions, warnings, and complications listed in the instructions. Patients were made aware that treatment with a gastric balloon could lead to complications, and severe complications might require endoscopic or surgical interventions. Patients were encouraged to maintain access to emergency medical facilities in case of developing complications during the treatment with Allurion’s ingestible gastric balloon.

### 2.2. Inclusion and Exclusion Criteria

The participant selection process was tailored toward adult patients aged 18 years and above who demonstrated a body mass index (BMI) of ≥27 kg/m^2^. In addition, these patients had previously undergone medical and nutritional counseling, and despite concerted efforts toward diet modification, increased exercise, and broader lifestyle changes, had failed to lose weight. Patients included in this study must have finalized the 16-week follow-up period after swallowing the gastric balloon. All participants had to use the Scale App Health Tracker, the home scale provided by the producer, and the provided smartwatch to measure their vitals.

The exclusion criteria were designed to negate potential confounders in this study. Patients with known contraindications to Allurion’s ingestible gastric balloon, such as a history of gastric surgery or the presence of a large hiatal hernia, were excluded. Further, patients with severe comorbid conditions like advanced cardiac, hepatic, or renal disease, or those unable or unwilling to adhere to the study protocol, were excluded. Additionally, patients with a history of diagnosed eating disorders were also excluded from this study.

**Figure 1:**
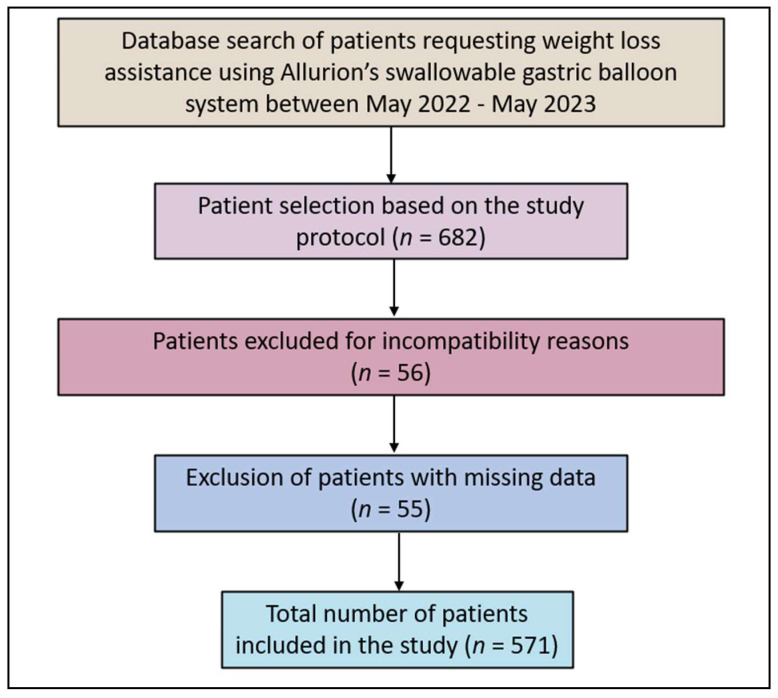
Retrospective study flowchart.

After the selection of 682 patients who used Allurion’s ingestible gastric balloon, a total of 111 were excluded due to incomplete data or other incompatibility reasons as per the study selection protocol or were lost to follow-up. A total of 571 patients were selected and subsequently enrolled in this study, as presented in Figure 1.

### 2.3. Study Variables, Definitions, and Procedures

Primary variables included baseline parameters, such as age, initial weight, and BMI. The secondary variables encompassed a more extensive array of metrics, including weight progress, the qualitative activity level delineated as less active or more active, the type of physical activity, such as running, cycling, football, walking, or hiking (as self-reported by the patients during the follow-up period), the duration of physical activity (as a self-reported measurement of a certain physical activity), a dual qualitative and quantitative sleep analysis, daily step count (measured on the Allurion Technologies Inc. smartwatch that was worn by all study participants), and an exhaustive body composition analysis featuring body fat, lean mass, bone mass, body water, and visceral fat, which were measured with the Allurion Technologies Inc. scale. Sequential changes in these parameters over the follow-up period were also documented.

Considering the retrospective nature of this study, patients were grouped based on activity level after the intervention and after reviewing their daily activity. To categorize patients into “Less Active” and “More Active” groups for our analyses, we established a cutoff based on the median number of steps taken per day, which in our study, was 8288 steps. This approach is consistent with the median split method, which is a standard statistical technique used for dichotomizing continuous variables to create comparable groups within a population [34]. Moreover, this threshold aligns with the existing literature indicating that a range of 7000 to 10,000 steps per day represents moderate-intensity physical activity for adults, with higher counts suggestive of greater activity levels [35].

To facilitate continuous monitoring and data recording, participants were provided with a comprehensive digital health suite, consisting of the Scale App Health Tracker installed on their personal smartphones and a smartwatch to record vitals like heart rate and sleep patterns in real time. Furthermore, a home-use body weight scale was distributed among the participants for daily weight measurements. All patients received the same weight scale and smartwatch models by Allurion Technologies Inc. The frequency of patients’ symptoms was recorded after being self-reported during the follow-up time.

The Allurion system was composed of the Allurion device, which became the Allurion balloon when filled, and the Allurion filling set. The Allurion device was a gastric balloon enclosed in a capsule, which was swallowed by the patient to introduce it into the stomach. Once confirmed in the stomach, the balloon could be filled using the provided filling kit. The filled balloon remained in the stomach for approximately 16 weeks, promoting satiety and reducing food intake. At the end of the treatment period, the device was designed to automatically open and empty itself, allowing it to pass through the gastrointestinal tract for excretion. The procedure was performed by qualified healthcare professionals under the supervision of a trained physician.

The physicians ensured that patients had access to endoscopy in case intervention to puncture or remove the device was necessary. They also provided patients with access to a supervised nutrition program. The medical professionals performing the procedure were certified or trained in using the Allurion gastric balloon system before placing the device. Physicians were adequately trained and qualified to perform the procedure using the Allurion gastric balloon system. They possessed the necessary knowledge of gastrointestinal anatomy and the safe and appropriate use of the device. Additionally, they were aware of potential complications and were prepared to handle any emergencies that might arise during or after the procedure. The procedure was performed in hospital settings, where emergency endoscopy could be performed in case of emergency during and after the device was swallowed.

To perform the procedure, physicians needed proper certification or training in using the Allurion gastric balloon system [36]. They were knowledgeable about gastrointestinal anatomy, endoscopy techniques, and the safe use of the device. Additionally, they were prepared to handle potential complications and emergencies that could occur during the procedure or treatment period. The Allurion training capsule is recommended to be swallowed before placing the device. It is prepared for ingestion by the patient after removal from its packaging. The patient is instructed to swallow the capsule with water, and if they have difficulty swallowing it, they can use a stiletto to assist in the process.

Before placing the device, patient preparation is performed by refraining from consuming solid foods for at least 8 h and liquids for at least 2 h. The device is then filled with fluid using an infuser and a specific procedure. For the placement of the device in the stomach, the patient is ideally seated. The capsule is placed on the back of the tongue, and the patient is asked to swallow it with water. If the capsule cannot be swallowed within 3 min, an alternative method involving the use of a stiletto is used. The proper placement of the device in the stomach is confirmed using fluoroscopy and/or abdominal X-rays. Once confirmed, the device is filled with fluid through a specified process involving pressure monitoring and adjustment. If necessary, the device can be removed endoscopically under anesthesia. Endoscopic removal involves the aspiration of fluid from the device and extracting the deflated device through the mouth using a specialized needle and grasping device.

### 2.4. Statistical Analysis

Data management and analysis were conducted utilizing the statistical software SPSS version 24.0 (SPSS Inc., Chicago, IL, USA). For analysis, patients were enrolled in two study groups based on their level of activity (less active and more active). The Kolmogorov–Smirnov test was used to determine the normality of data. Normally distributed variables were represented as mean ± standard deviation (SD), while non-parametric continuous variables were represented with the median value and the interquartile range (IQR). A Mann–Whitney U-test was performed to compare the median values of non-normally distributed continuous variables. To compare the means of normally distributed data before and after the intervention, paired *t*-tests were employed (for comparing same-group data), and independent *t*-tests were used to compare different groups. The chi-square test was utilized for comparing proportions. The ANOVA test was used to compare two or more means, and a mixed linear model was used to analyze the dataset in terms of group interactions. A *p*-value threshold of less than 0.05 was set for statistical significance. All results were double-checked to ensure accuracy and reliability.

## 3. Results

### 3.1. Patients’ Characteristics

As described in Table 1, a total of 571 patients were included in this study, with a median age of participants of 41.0 years and an age range spanning from 20 to 71 years. Patients above 60 years constituted the smallest age category, at 8.6%. Nausea (3.7%) and abdominal discomfort (3.5%) were the most commonly reported symptoms.

In terms of weight metrics, the starting weight of all participants was at a mean of 97.9 kg. The mean BMI in the less active group was 33.9 kg/m^2^, while 32.5% had a BMI ranging from 30 to 35 kg/m^2^. The more active patients had a mean starting weight of 97.7 kg and a mean starting BMI of 35.2 kg/m^2^, with no significant differences between groups. Lastly, the activity level of participants was assessed based on the daily number of steps they took. The median daily step count stood at 8288, with an interquartile range spanning from 3617 to 12,959 steps.

The more active group had a notably higher proportion of individuals participating in running activities, with 55.8% compared to 32.5% in the less active group. Conversely, a more significant portion of the less active group participated in other forms of exercise (35.3%) not specified in the table, compared to only 13.0% in the more active group. Participation in cycling was slightly higher in the less active group (12.2%) than in the more active group (9.5%), while football saw similar participation rates between the groups, with 19.9% for the less active group and 21.8% for the more active group.

Regarding the duration of activity per session, a significant difference was documented, with a median time of 58.0 min for the more active group compared to 40.5 min for the less active group (*p*-value < 0.001), as presented in Table 2. Moreover, the minutes of moderate-intensity physical activity per week were 110 in the less active group versus 166 in the more active group. The more active group exhibited a considerably higher median step count per day, with 10,944 steps, in contrast to the less active group, which recorded a median of 6629 steps (*p*-value < 0.001).

### 3.2. Outcomes and Follow-Up

In analyzing the data from Table 3, it was noted that both the less active and more active groups demonstrated significant changes in various health metrics over the 4-month period post-intervention with Allurion’s ingestible gastric balloon. Both groups showcased a remarkable decrease in body fat percentage, with the less active group reducing from 33.1 ± 9.4% to 28.3 ± 10.2% and the more active group from 32.2 ± 10.9% to 27.5 ± 10.1%. These changes were statistically significant within each group, as evidenced by *p*-values less than 0.001. However, when comparing the changes between the groups, there was no significant difference in the body fat reduction observed between the less active and more active groups.

Lean mass experienced diverging trends in the two groups. The more active group saw a significant increase in lean mass from 50.9% to 53.7% (*p* = 0.022), while the less active group also increased but not significantly from 49.5% to 51.3% (*p* = 0.130). Comparing the post-intervention values between groups revealed a statistically significant difference with a *p*-value of 0.045.

In terms of bone mass, no significant changes were observed within either group, but interestingly, a comparison between the post-intervention values of the two groups showed a statistically significant difference with a *p*-value of 0.001. The body water percentage decreased significantly in the more active group from 44.6% to 42.1% (*p* = 0.003), whereas the less active group did not exhibit a significant change. The post-intervention comparison between groups did not show a significant difference with a *p*-value of 0.523.

A notable decrease was observed in visceral fat levels in both groups, with the more active group showcasing a more substantial reduction (*p* = 0.001) compared to the less active group (*p* = 0.029), but the comparison between groups post-intervention showed no significant difference (*p* = 0.711).

Both groups experienced a significant reduction in BMI and weight, as demonstrated by *p*-values less than 0.001. The less active group went from a BMI of 33.9 to 29.2 and weight from 98.2 kg to 84.6 kg. Similarly, the more active group reduced from a BMI of 35.2 to 30.4 and weight from 97.7 kg to 82.1 kg. The post-intervention comparison between groups, however, did not display a significant difference, with *p*-values of 0.229 and 0.158 for BMI and weight, respectively.

Over the course of 4 months, there was a notable decrease in the body fat percentage, from an initial 32.7% to 27.9% at the end of the study period, indicating a considerable decline. The *p*-value of less than 0.001 in this context signifies that this reduction was statistically significant. Concurrently, the lean mass exhibited a subtle yet statistically significant increase, growing from 49.8% to 52.6% (*p* = 0.008), as presented in Table 3. Similarly, bone mass underwent a slight modification over the span of 4 months, ranging from 3.1 initially to 3.2 toward the end, with a *p*-value of 0.039, confirming the statistical significance of this upward trend. Additionally, the body water percentage demonstrated a modest decrease, sliding from 44.7 to 42.5, a change that was statistically validated with a *p*-value of 0.009. Analyzing the basal metabolic rate, an upward trajectory was observed, with the rate augmenting from 1554.1 to 1662.0; the statistical significance of this uptrend was corroborated by a *p*-value of 0.024. Furthermore, visceral fat levels descended notably from 13.4 to 12.0 (*p*-value < 0.001).

**Figure 2:**
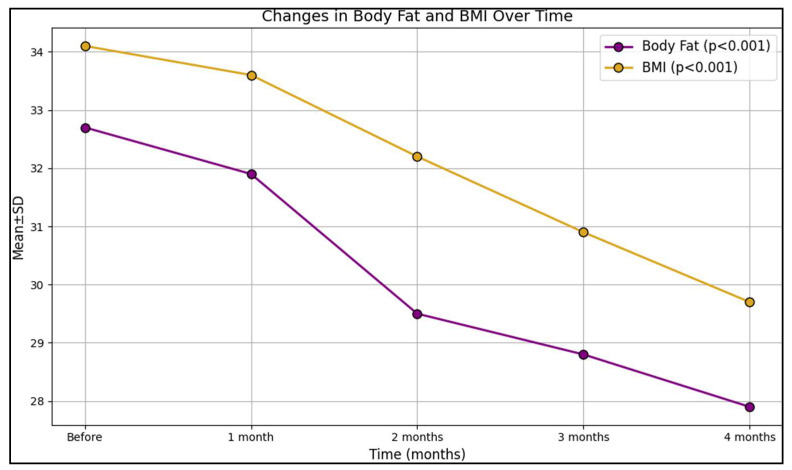
Changes in body fat and BMI with Allurion’s ingestible gastric balloon.

A pronounced reduction was observed in the BMI of the participants over the 4 months, which decreased from 34.1 to 29.7, a change that was statistically significant with a *p*-value less than 0.001, as seen in Figure 2. Correspondingly, the weight of the participants experienced a substantial decline, plummeting from 97.9 kg to 84.0, a change substantiated with a *p*-value less than 0.001, indicating its statistical significance.

Significant weight loss and body composition improvements were observed over a four-month period. Both the less active and more active groups saw marked reductions in body fat percentage. Specifically, the less active group’s body fat decreased from an initial 33.1% to 28.3%, while the more active group saw a reduction from 32.2% to 27.5%. Despite these changes, there was no significant Time*Group interaction, indicating that both groups benefited similarly from the treatment.

Lean mass increased modestly from 49.5 to 51.3 in the less active group and from 50.9 to 53.7 in the more active group, with a significant interaction suggesting that the more active group experienced greater gains. Bone mass remained stable, with a slight increase in the more active group from 3.2 to 3.3, and a significant Time*Group interaction highlighted varying trends between groups. Both BMI and weight significantly decreased in all participants, with the less active group’s BMI reducing from 33.9 to 29.2 kg/m^2^ and weight from 98.2 to 84.6 kg, demonstrating the treatment’s effectiveness across varying levels of activity, as described in Table 4.

## 4. Discussion

### 4.1. Literature Findings

Our comprehensive study aimed to evaluate the short-term efficacy and patient engagement of Allurion’s ingestible gastric balloon using the Scale App Health Tracker. This study reveals pivotal insights into the characteristics and outcomes of patients undergoing a 4-month intervention with Allurion’s ingestible gastric balloon. The baseline characteristics demonstrate a predilection toward middle-aged individuals, with a significant proportion falling within the “obese” category as per BMI classifications. Notably, the average participant embarked on this study at a significant weight and BMI, indicating a pertinent necessity for the intervention. This population distribution elucidates the critical significance of targeting interventions at individuals in these age and BMI groups, potentially harboring a higher risk for health complications associated with obesity.

Delving into the activity levels, a clear demarcation between the less active and more active groups emerges, particularly concerning age and the types of exercises. The younger demographic in the more active group possibly underscores a generational inclination toward maintaining a higher physical activity level. Furthermore, the types of exercise and the durations per session were significantly different between the groups. This observation merits further exploration into the motivations and barriers to different forms of physical activity across diverse age groups, possibly paving the way for more nuanced, age-specific intervention strategies.

The observed outcomes offer a nuanced view of the intervention’s efficacy. The intervention evidently fostered substantial improvements in several health metrics across both groups. Notably, both groups exhibited a significant reduction in body fat percentage, an outcome that underscores the effectiveness of the intervention. However, the nuances between groups in terms of lean mass increment and visceral fat reduction indicate differential benefits, possibly pointing toward the added advantage of higher physical activity levels in augmenting the positive effects of the intervention. Moreover, the lack of significant differences in the post-intervention comparison of several metrics between the groups hints at the overarching effectiveness of the intervention, regardless of the pre-existing activity levels.

Furthermore, the data point toward diverging trends in lean mass and bone mass between the two groups. The more active group not only exhibited a significant increase in lean mass but also depicted significant variations in bone mass post-intervention. This observation might indicate the supplementary benefits of heightened physical activity in fostering positive adaptations in body composition, beyond what is achievable through weight loss interventions alone. This disparity necessitates further research to untangle the potential synergistic effects of increased physical activity and weight loss interventions on lean and bone health.

The placement of intragastric balloons (IGB) has been shown to not only aid in effective weight loss but also significantly improve obesity-related comorbid conditions. Several studies indicate marked improvements in conditions like diabetes mellitus, hypertension, and dyslipidemia following IGB placement, with some patients requiring reduced medication dosages or less aggressive treatment methods [37,38]. There is evidence that NAFLD and respiratory disorders, like obstructive sleep apnea, also improve after IGB insertion, supported by enhancements in liver function tests and lung function parameters [39,40]. Obesity often results in a low-grade inflammatory state due to adipose tissue remodeling, which can lead to metabolic disorders. Fortunately, weight reduction from IGB can potentially mitigate this inflammatory state, thereby reducing the risk of cardiovascular events. A study involving 42 patients found that IGB placement over six months reduced inflammation markers and improved the metabolic profile [41]. However, despite various studies on weight loss, data on the metabolic advantages of IGBs remain somewhat scarce. A comprehensive meta-analysis revealed that IGB significantly improved metabolic parameters like fasting blood glucose and hemoglobin A1c [42]. As such, IGB can serve as an additional treatment option in a multidisciplinary strategy for managing obese patients with metabolic syndrome.

Several other studies were performed using this device or similar devices, thus allowing for the establishment of a comprehensive understanding of Allurion’s ingestible gastric balloon’s performance and implications in weight management. In one significant study involving a large, multicenter population, Allurion’s ingestible gastric balloon demonstrated a noteworthy safety profile, coupled with an excellent efficacy record [27]. This study analyzed data from 1770 patients and reported weight loss results at the four-month mark, which included a 14.2% total body weight loss (TBWL) and an impressive 67.0% excess weight loss (EWL). All the metabolic parameters, including triglycerides, LDL cholesterol, and HbA1c, showcased improvement post-treatment. Although the procedure’s safety was highly praised, this study reported a small fraction of complications, including spontaneous hyperinflations, esophagitis, pancreatitis, gastric dilation, gastric outlet obstruction, and even a case of gastric perforation. Nevertheless, this study provides additional information about patient outcomes and laboratory parameters that were not identified in our research.

Another study that evaluated the effectiveness of the swallowable intragastric balloon combined with lifestyle coaching emphasized the combination’s potential benefits [43]. With a patient pool of 336 individuals, the results demonstrated that the treatment led to an average total weight loss of 11.0% after a year. Notably, the majority of the patients reported temporary symptoms like nausea and gastric pain, which typically resolved within a week. The inclusion of a 12-month coaching program also underscores the significance of combining medical interventions with behavioral strategies to enhance the treatment’s overall effectiveness and patient adherence.

Moreover, a major study underlined the efficacy and safety of Allurion’s ingestible gastric balloon via a meta-analysis [44]. Analyzing six different studies, the meta-analysis found that the early removal rate was 2.3%, with an overall %TWL at 4–6 months of 12.8%. By the end of one year, the %TWL was higher than 10%. In our study, the overall BMI decrease was 12.9%, and there was a 14.2% decrease in the total weight, which is in accordance with the producer’s data. While the efficacy seems promising, the meta-analysis reported severe adverse events, albeit rare, highlighting the importance of patient monitoring and swift medical interventions. However, in our study, the removal rate was only four patients (0.7%) for intolerance or hyperinflation, while we did not identify any cases of more severe complications, such as gastric outlet obstruction.

Endoscopically placed intragastric balloons have been at the forefront of obesity treatments for over three decades, as noted in a review of various balloon types [45]. While these balloons, including Allurion’s ingestible gastric balloon, have bridged the gap between lifestyle modifications and surgical interventions effectively, there are indications that their sustainable effectiveness can be limited, as some studies report that patients return to their pre-treatment weight after balloon removal. This raises questions regarding the long-term viability of such treatments without the integration of continuous post-treatment support mechanisms. In addition, several potential complications may occur during the ingestion of the balloon or while the device is located in the stomach, such as gastric outlet obstruction [46].

Therefore, Allurion’s gastric balloon has emerged as a promising tool in the fight against obesity, providing significant short-term weight loss. However, its efficacy can vary based on individual response and adherence to post-treatment guidelines. Moreover, while the safety profile is largely commendable, the potential risks and complications cannot be overlooked. Ensuring the continuous monitoring of patients and integrating behavioral strategies, as seen with the combined lifestyle coaching, may enhance the overall treatment outcomes and patient engagement.

### 4.2. Study Limitations

This study had several limitations. First, this study was a single-center, observational study, which may limit the generalizability of the findings to a broader population. This study included 571 patients, which, although comprehensive, may not capture the full spectrum of responses to Allurion’s ingestible gastric balloon across different demographics and geographic locations. Second, this study relied on the Scale App Health Tracker for monitoring patient engagement; however, the reliance on digital health technology might introduce bias as it assumes that all participants are equally comfortable and adept at using digital devices, and it may not capture the full nuances of patient engagement and quality of life changes that could be obtained through more qualitative methods. Moreover, indirect assessment of QoL through weight loss is an inherent limitation. This approach may not capture the multifaceted aspects of QoL that can indeed diverge from the outcomes related to weight loss. Future prospective studies should consider incorporating direct measures of QoL to provide a more comprehensive understanding of how weight management interventions impact overall well-being. Also, data were collected from at-home scales, which brings limitations in terms of accuracy and standardized measurement practices, compared to in-clinic measures. Third, this study’s duration was 16 weeks, for the duration of the balloon residency time in the stomach, which, while sufficient to assess short-term efficacy and patient engagement, does not provide insights into the long-term effects and engagement associated with Allurion’s ingestible gastric balloon. Fourth, this study excluded patients with severe comorbid conditions, a history of gastric surgery, large hiatal hernia, or diagnosed eating disorders, which means the findings may not be applicable to these groups of patients. Another possible limitation is the lack of data regarding the daily calorie intake of patients, which can influence significantly their weight loss progress, regardless of activity level. Finally, this study did not include a control group of patients not receiving Allurion’s ingestible gastric balloon, which makes it difficult to assess the effects of the intervention relative to a standard treatment or no treatment at all.

## 5. Conclusions

This study validates Allurion’s ingestible gastric balloon as an effective non-invasive method for immediate weight loss, applicable across varying activity levels. Significant weight reduction was achieved in both less active and more active patients, with no notable differences in outcomes between them. This intervention emerges as a promising tool for obesity management, warranting further exploration into its long-term effects post-elimination.

## Figures and Tables

**Table 1:** Background characteristics of the study cohort.

Variables	Total (n = 571)	Less Active (n = 286)	More Active (n = 285)	*p*-Value
Age, years (median, IQR)	41.0 (33.5–48.5)	43.5 (38.7–47.3)	40.3 (35.3–47.3)	<0.001
Age range (years)	20–71	24–71	20–68	–
Age category, (n,%)				<0.001
20–40 years	230 (40.3%)	86 (30.1%)	144 (50.5%)	
40–60 years	292 (51.1%)	173 (60.5%)	119 (41.8%)	
>60 years	49 (8.6%)	27 (9.4%)	22 (7.7%)	
Starting weight, kg (mean ± SD)	97.9 ± 22.3	98.2 ± 22.8	97.7 ± 21.0	0.842
Starting BMI (kg/m^2^) (mean ± SD)	34.5 ± 10.1	33.9 ± 10.3	35.2 ± 9.6	0.181
BMI category (kg/m^2^), (n,%)				0.106
27–30	118 (20.7%)	54 (18.9%)	64 (22.5%)	
30–35	175 (30.6%)	93 (32.5%)	82 (28.8%)	
35–40	97 (16.9%)	57 (19.9%)	40 (14.0%)	
>40	181 (31.8%)	82 (28.7%)	99 (34.7%)	
Activity level, number of steps (median, IQR) *	8288 (3617–12,959)	6629 (4438–12,120)	10,944 (8361–13,527)	<0.001
Symptoms after balloon ingestion, (n,%)				0.479
No symptoms	547 (95.8%)	257 (89.9%)	257 (90.2%)	
Nausea	21 (3.7%)	9 (3.1%)	12 (4.2%)	
Vomiting	16 (2.8%)	7 (2.4%)	9 (3.2%)	
Abdominal discomfort	20 (3.5%)	13 (4.5%)	7 (2.5%)	

*—Measured based on the daily number of steps; IQR—Interquartile range; SD—Standard Deviation.

**Table 2:** Patients’ physical activity.

Variables	Less Active (n = 286)	More Active (n = 285)	*p*-Value
Type of exercise * (n,%)			<0.001
Running	93 (32.5%)	159 (55.8%)	
Cycling	35 (12.2%)	27 (9.5%)	
Football	57 (19.9%)	62 (21.8%)	
Other	101 (35.3%)	37 (13.0%)	
Minutes of activity per session (median, IQR)	40.5 (31.0–54.5)	58.0 (50.5–66.0)	<0.001
Number of daily steps (median, IQR)	6629 (4438–12,120)	10,944 (8361–13,527)	<0.001
Minutes of moderate-intensity physical activity per week (median, IQR)	110 (84–136)	166 (121–184)	<0.001

*—Except for walking; IQR—interquartile range.

**Table 3:** Follow-up results among all study participants.

Variables (Mean ± SD)	Before	1 Month	2 Months	3 Months	4 Months	*p*-Value *
Body fat	32.7 ± 11.3	31.9 ± 12.0	29.5 ± 13.2	28.8 ± 10.8	27.9 ± 12.6	<0.001
Lean mass	49.8 ± 14.9	50.1 ± 16.3	50.4 ± 13.8	51.5 ± 14.2	52.6 ± 15.4	0.008
Bone mass	3.1 ± 0.8	3.1 ± 0.8	3.0 ± 1.0	3.1 ± 1.2	3.2 ± 1.4	0.039
Body water	44.7 ± 11.9	44.9 ± 12.7	44.0 ± 13.3	43.1 ± 14.6	42.5 ± 13.8	0.009
Visceral fat	13.4 ± 5.9	13.0 ± 6.4	12.8 ± 6.1	12.3 ± 6.3	12.0 ± 6.8	0.001
BMI (kg/m^2^)	34.1 ± 7.8	33.6 ± 8.0	32.2 ± 9.5	30.9 ± 10.3	29.7 ± 12.5	<0.001
Weight (kg)	97.9 ± 21.8	93.0 ± 19.6	89.6 ± 22.7	86.1 ± 23.7	84.0 ± 23.9	<0.001

*—Compared using the ANOVA test; SD—Standard deviation; BMI—Body mass index.

**Table 4:** Analysis of health parameters during the four-month intervention.

Outcome	Group	Baseline (Mean ± SD)	Month 1 (Mean ± SD)	Month 2 (Mean ± SD)	Month 3 (Mean ± SD)	Month 4 (Mean ± SD)	*p*-Value for Time * Group Interaction	*p*-Value for Group Main Effect	*p*-Value for Time Main Effect
Body fat	Less Active	33.1 ± 9.4	32.5 ± 9.2	31.8 ± 9.0	29.7 ± 9.8	28.3 ± 10.2	0.346	0.462	<0.00001
	More Active	32.2 ± 10.9	31.8 ± 10.7	31.2 ± 10.4	29.1 ± 10.0	27.5 ± 10.1			
Lean mass	Less Active	49.5 ± 14.8	50.0 ± 14.6	50.4 ± 14.3	50.9 ± 13.9	51.3 ± 13.6	0.045	0.309	0.022
	More Active	50.9 ± 14.2	51.4 ± 14.0	51.9 ± 13.8	52.5 ± 13.5	53.7 ± 15.0			
Bone mass	Less Active	3.1 ± 0.8	3.0 ± 0.9	2.9 ± 0.9	2.9 ± 1.0	3.0 ± 1.0	0.001	0.374	0.039
	More Active	3.2 ± 1.0	3.1 ± 1.1	3.0 ± 1.2	3.2 ± 1.1	3.3 ± 1.2			
Body water	Less Active	43.9 ± 10.5	44.1 ± 10.2	44.3 ± 9.9	43.2 ± 10.8	42.7 ± 11.9	0.523	0.289	0.009
	More Active	44.6 ± 9.3	44.8 ± 9.0	45.0 ± 8.7	43.7 ± 9.6	42.1 ± 10.5			
Visceral fat	Less Active	13.0 ± 5.4	12.7 ± 5.1	12.4 ± 4.9	12.1 ± 5.3	11.9 ± 6.6	0.711	0.254	0.001
	More Active	13.8 ± 6.0	13.4 ± 5.7	12.9 ± 5.5	12.3 ± 5.1	12.1 ± 6.3			
BMI (kg/m^2^)	Less Active	33.9 ± 10.3	33.5 ± 10.1	33.1 ± 9.9	30.6 ± 11.2	29.2 ± 11.8	0.229	0.181	<0.00001
	More Active	35.2 ± 9.6	34.8 ± 9.4	34.4 ± 9.1	31.7 ± 11.5	30.1 ± 12.0			
Weight (kg)	Less Active	98.2 ± 22.8	96.9 ± 22.1	95.6 ± 21.4	92.8 ± 20.6	84.6 ± 19.3	0.158	0.842	<0.00001
	More Active	97.7 ± 21.0	96.3 ± 20.3	95.0 ± 19.6	90.4 ± 21.1	82.1 ± 22.9			

*—Compared using the ANOVA test; SD—Standard deviation; BMI—Body mass index.

## Data Availability

Data are available on request.
